# The African Cichlid Fish *Astatotilapia burtoni* Uses Acoustic Communication for Reproduction: Sound Production, Hearing, and Behavioral Significance

**DOI:** 10.1371/journal.pone.0037612

**Published:** 2012-05-18

**Authors:** Karen P. Maruska, Uyhun S. Ung, Russell D. Fernald

**Affiliations:** Department of Biological Sciences, Stanford University, Stanford, California, United States of America; Claremont Colleges, United States of America

## Abstract

Sexual reproduction in all animals depends on effective communication between signalers and receivers. Many fish species, especially the African cichlids, are well known for their bright coloration and the importance of visual signaling during courtship and mate choice, but little is known about what role acoustic communication plays during mating and how it contributes to sexual selection in this phenotypically diverse group of vertebrates. Here we examined acoustic communication during reproduction in the social cichlid fish, *Astatotilapia burtoni*. We characterized the sounds and associated behaviors produced by dominant males during courtship, tested for differences in hearing ability associated with female reproductive state and male social status, and then tested the hypothesis that female mate preference is influenced by male sound production. We show that dominant males produce intentional courtship sounds in close proximity to females, and that sounds are spectrally similar to their hearing abilities. Females were 2–5-fold more sensitive to low frequency sounds in the spectral range of male courtship sounds when they were sexually-receptive compared to during the mouthbrooding parental phase. Hearing thresholds were also negatively correlated with circulating sex-steroid levels in females but positively correlated in males, suggesting a potential role for steroids in reproductive-state auditory plasticity. Behavioral experiments showed that receptive females preferred to affiliate with males that were associated with playback of courtship sounds compared to noise controls, indicating that acoustic information is likely important for female mate choice. These data show for the first time in a Tanganyikan cichlid that acoustic communication is important during reproduction as part of a multimodal signaling repertoire, and that perception of auditory information changes depending on the animal's internal physiological state. Our results highlight the importance of examining non-visual sensory modalities as potential substrates for sexual selection contributing to the incredible phenotypic diversity of African cichlid fishes.

## Introduction

Courtship and mating involves the production of sexual signals that convey crucial information on the senders' identity, quality, motivation, readiness, and social status. Reception of this information by an intended receiver must then be integrated with the animals' internal state and translated into adaptive behaviors. Importantly, many animals use multiple sensory modalities during reproductive interactions, where each sensory channel may provide a different type of information to an intended receiver [1], [2], [3], [4]. Accounting for this complex multimodal communication is essential for understanding how mate choice decisions are made and how this might influence sexual selection [5]. However, the role of multimodal communication in mating decisions is sorely understudied across taxa [6], especially in fishes [7], [8], [9], which represent by far the largest and most reproductively diverse group of vertebrates.

East African cichlid fishes use multiple senses (i.e., visual, chemosensory, acoustic, mechanosensory) to coordinate their complex social behaviors [9]. Moreover, their adaptive radiation and rapid speciation is unparalleled among vertebrates [10], [11], making this group of fishes excellent models to examine the role of multimodal communication in sexual selection. Due to the diversity in bright nuptial coloration patterns among cichlids, the role of the visual system as a substrate for sexual selection has received considerable attention [9], [10], [12], [13], [14], [15], [16], while the impact of other senses such as the auditory system remain relatively unexplored [9], [17]. Importantly, however, recent analyses indicate that visual communication alone is not sufficient to explain the diversity of African cichlids [10], [18], suggesting that other forms of sensory communication may play significant roles in mate choice. For example, differences in male courtship sounds among sympatric cichlid species in Lake Malawi are consistent with the hypothesis that acoustic signaling may contribute to reproductive isolation and speciation [17], [19], [20], but whether females are physiologically capable of distinguishing these signal differences among species is not known. While courtship sounds have been described in many different cichlids, representing both rift lake and riverine species [17], [21], [22], [23], [24], little is known about their hearing abilities, how sounds are matched to their auditory capabilities, and what role acoustic signaling might have during female mating decisions (but see [25]). Importantly, none of this information (sound production, hearing ability, biological function) is collectively available for a single cichlid species. Further, the perception of auditory information can be profoundly influenced by an animals' physiological state, such as reproductive condition, neuropeptide levels in the brain, and circulating levels of sex- and stress-related steroid hormones [26], [27], [28], [29], [30], [31], suggesting that internal cues can modulate how individuals respond to acoustic signals. To fully appreciate how females make mate choice decisions, it is crucial to understand all of the signaling systems that contribute to neural computations resulting in adaptive behaviors. These insights may also guide our understanding of how different signaling systems have evolved within a species flock.

To address questions on the role of multimodal communication during reproduction, we use the African cichlid fish *Astatotilapia burtoni* as a model. This species is endemic to shallow shore pools of Lake Tanganyika, the geologically oldest lake in the rift valley system of East Africa, where males exist in one of two reversible phenotypes: 1) dominant territorial males (∼10–30% of population) that are brightly colored, aggressively defend a spawning territory, and actively court and spawn with females; and 2) subordinate non-territorial males that school with and resemble females in coloration, perform submissive behaviors, do not typically court females, and are reproductively suppressed [32]. Males can and do reversibly switch between dominant and subordinate phenotypes depending on the composition of the social environment, and this social transformation causes a suite of behavioral and physiological changes in the brain and along the reproductive axis [33], [34]. *Astatotilapia burtoni* lives in a lek-like social system where dominant males perform behavioral courtship displays to entice passing females into their territories to spawn. After spawning, females rear the developing young in their mouths (mouthbrooding) for ∼2 weeks before releasing them, and then will recover physiologically for several weeks before spawning again [35], [36]. While visual cues are essential for social behaviors in this species [32], [37], there is also evidence for the importance of chemosensory and acoustic signals during mating [38], [39], [40], [41]. However, while sound production was examined previously in *A. burtoni* [23], [39], [40], [42], a detailed analysis of the characteristics of courtship-specific sounds and associated visual behaviors was not performed, nor was hearing ability or the biological significance of acoustic communication during reproduction investigated.

The overall goal of this study was to determine the importance of acoustic communication during courtship and reproduction in a highly social, and notably visual, African cichlid fish. Specifically, we characterized the sounds and associated behaviors produced by dominant males during courtship, tested whether there were differences in hearing ability associated with female reproductive state or male social status, and then tested the hypothesis that female mate preference is influenced by male sound production. Unlike most previous studies in fish bioacoustics that conduct an in-depth examination of one particular aspect of communication (e.g., sound production *or* hearing ability), we chose a more inclusive approach and focused on a single behavioral context (courtship) to examine acoustic signaling from both sender and receiver perspectives. To our knowledge, this is the first study to simultaneously describe sound production, hearing ability, and behavioral significance of acoustic communication during courtship in a single fish species, and to show non-seasonal reproductive state changes in hearing abilities correlated with circulating sex-steroid levels. Our results support the hypothesis that acoustic signaling is an important sensory channel in the natural courtship repertoire of *A. burtoni*, and highlight the importance of examining non-visual sensory modalities used during social interactions as potential substrates for sexual selection contributing to the remarkable phenotypic diversity of cichlid fishes.

## Methods

### Animals

Adult laboratory-bred cichlid fish *A. burtoni* were derived from wild-caught stock in Lake Tanganyika, Africa, and mixed-sex community groups were maintained in aquaria under environmental conditions that mimic their natural habitat (28°C; pH 8.0; 12 h light∶12 h dark full spectrum illumination; constant aeration). Aquaria contained gravel-covered bottoms with half terra cotta pots that served as shelters and spawning territories. Fish were fed cichlid flakes and pellets (AquaDine, Healdsburg, CA, USA) each morning. All experimental procedures were approved by the Stanford Administrative Panel for Laboratory Animal Care (#A3213-01).

### Courtship sound recordings and analysis of sound characteristics

To determine whether males produced sounds during typical courtship behaviors (e.g., body quivers, leading, tail waggles, pot entries), we placed a single dominant reproductively active male in the center compartment of an experimental tank (48×165×30 cm) along with three females and a single terra cotta pot to serve as a territory. This central compartment (48×30 cm) was bordered on either side by larger community tanks that contained fish of both sexes and various reproductive states so that the subject male could interact visually, but not physically, with his neighbors across a clear acrylic barrier. The subject male (*N* = 22 males total) was allowed to establish a territory and acclimate for 24 hrs prior to sound recordings. To examine possible relationships between sound characters and male body size, we also used dominant males that ranged in size from 47–87 mm standard length. These dominant males were selected from community tanks where they were verified to hold a territory and perform typical dominance behaviors [43], [44] for 3–4 wks prior to testing.

On the day of the experiment, a calibrated hydrophone (HTI-94, High Tech, Inc., Gulfport, MS., USA; sensitivity −163.7 dB re: 1 V/µPa; frequency response 2 Hz–30 kHz) was suspended near the pot shelter in the center of the experimental tank and attached to the external microphone input of a digital video camera (Canon FS20) that was positioned directly in front of the tank to record behaviors for later analysis. The resident females were then removed from the subject male's compartment, replaced with 5–6 gravid (reproductively receptive) females, and the behaviors and associated sound production of the subject male was recorded for 20–30 min. Video files were then analyzed for the following measures: time of sound production, time of behavioral quiver display, and percentage of quivers associated with sound production.

To characterize the courtship sounds produced by males, acoustic channels recorded from the hydrophone were exported from the video files and analyzed (Cool Edit Pro v2.1, Syntrillium software). Sound files were down-sampled (6000 Hz sample rate, no aliasing) and filtered (FFT, filter size 7680, Hanning window, band-pass 60–3000 Hz) to remove low and high frequency noise in the recording room that could not be eliminated otherwise. The following measurements were performed on the waveforms for each individual courtship sound: total sound duration (ms), pulse duration (ms), number of pulses per sound, and interpulse interval (ms). Peak frequency (Hz) for each pulse within a sound train was calculated with a 128-point FFT (Hanning window). Only those waveforms that were clearly distinguishable above background noise were used in analyses (3–6 sounds per individual male). Source levels were not determined in this study because sounds were recorded directly on a video camera with unknown gain in order to synchronize the behavior and sound recordings. In this study, we did not put fish into social situations designed to examine male sound production in other behavioral contexts (e.g., territorial or agonistic interactions), nor did we test whether or not females also produced sounds in any context. We are confident that the sounds we recorded and analyzed were produced by the dominant subject males because they were only associated with male body quiver behaviors, relative sound intensity was lower with increasing distance between the quivering male and the hydrophone, and similar sounds were not recorded from all female groups that were visually exposed to a courting male.

### Hearing ability: auditory evoked potential (AEP) experiments

To determine *A. burtoni* hearing thresholds across frequencies, and to compare hearing abilities between sexes and between different reproductive states (for females) and social status (for males), we used the auditory evoked potential (AEP) technique. The AEP method is a minimally invasive electrophysiological technique that measures the electrical activity induced in the body tissues above the cranium as a proxy of overall brain activation evoked by sound playback, and is a common tool for determining hearing thresholds in fishes [45], [46], [47], [48], [49], [50], [51]. We tested hearing thresholds in subordinate (SL = 65±2.8 mm; BM = 7.6±0.91 g; *N* = 8) and dominant (SL = 61±1.0 mm; BM = 6.4±0.26 g; *N* = 8) males, and mouthbrooding (SL = 54±1.7 mm; BM = 3.5±0.34 g; *N* = 8) and gravid (SL = 55±1.7 mm; BM = 4.9±0.45 g; *N* = 8) females. Subordinate and dominant males were generated as previously described [39], and daily observations were made to verify that each individual maintained his social status for 4–5 wks prior to testing. We used mouthbrooding females that had been brooding for ∼2 wks, which had fully developed fry that were removed from the mouth just prior to recordings. Gravid females were initially chosen based on distended abdomens typically indicative of large ovaries, and were then verified to contain large and readily released oocytes at the end of the experiment. Gravid females with gonadosomatic index [GSI = (gonad mass/body mass)×100] values≤6.0 were excluded from analyses.

The AEP experimental setup, procedures, and threshold determinations were similar to those described previously [46]. Fish were briefly anesthetized in ice-cold tank water and benzocaine (0.1%), immobilized with an intramuscular injection of pancuronium bromide (∼0.0005–0.001 mg g^−1^ BM; Sigma, Inc.), and lightly restrained in a mesh harness with a clamp suspended from a PVC frame around the experimental tank. A gravity-fed water system with a tube placed in the mouth was used to ventilate the fish during all experiments. The circular experimental tank (36.5 cm high, 30 cm diam.) was placed on a vibration isolation platform and the fish was suspended in the center so that it was positioned 4–5 cm beneath the water surface and 14 cm above an underwater speaker (UW-30, Electro-Voice, Burnsville, MN; frequency response, 100–10,000 Hz) that was partially buried in gravel at the bottom of the tank. Recording electrodes (stainless-steel sub-dermal electrodes, Rochester Electro-Medical, Inc., Tampa, FL) were sealed on the ends with nail polish so that ∼1 mm of metal was exposed at the tip. The recording electrode was positioned in the dorsal musculature along the midline and directly above the braincase in the region of the medulla, a reference electrode was placed beneath the skin between the eyes, and a ground wire was placed in the tank water.

Sound stimuli were generated with a Cambridge Electronics Design (CED) Micro3 1401 system controlled by Spike 2 software and a CED 3505 attenuator, amplified (TOA CA-160), and sent to the underwater speaker. The following 11 frequencies were tested for each fish: 100, 200, 300, 400, 500, 600, 700, 800, 1100, 1500, and 2000 Hz. Stimuli consisted of 2000 repetitions of 20 ms pulses (for ≥200 Hz, 10 ms plateau, 5 ms rise and fall times; for 100 Hz, 10 ms plateau, 10 ms rise and fall times) with an interpulse interval of 100 ms, and stimulus artifacts in the AEP recordings were minimized by sequential alternation of pulse phase. For each test frequency, sounds were first presented at suprathreshold intensity and then decreased in 5 dB steps until an AEP response was no longer observed and threshold was determined (described below). Sound levels produced by the speaker were calibrated by placing a hydrophone (High Tech, Inc.) in the experimental tank at the position normally occupied by the fish head, presenting the sound stimuli (without phase alternation), and measuring the rms voltage at each test frequency and intensity. Sound pressure levels (SPL) were then determined according to Davidson et al. [52] with the following equation: SPL (dB_rms_ re: 1 µPa) = 20log_10_ (((*X*×10^3^)/HCV)×10^6^), where *X* is the rms voltage in mV and HCV (hydrophone calibration value) = 6531 V/µPa. While future experiments are needed to characterize the sound stimuli in terms of particle motion, for the purposes of this study, the measurement of hearing thresholds referenced to sound pressure alone provides a sufficient representation of the audiogram shape and *relative* differences in hearing thresholds between reproductive states and social status in this species.

Auditory evoked potentials recorded from the fish were differentially amplified (10,000×) and filtered (1–10,000 Hz) on a Brownlee amplifier (Model 440, Brownlee Precision Co., San Jose, CA.), and then digitized on a CED micro3 1401 system running Spike 2 software and stored on computer. For each sound intensity and test frequency, a total of 2000 repetitions were averaged to produce the AEP waveform response. Power spectrum analyses (FFT, 512 or 1024 points) were performed in Spike 2 on these averaged waveforms to examine for peaks at twice the stimulus frequency that result from the opposite orientation of hair cells in the sensory macula and non-linearities in the auditory system [53]. Threshold at each frequency was determined by both the averaged AEP trace and power spectrum and defined as the lowest sound level to show a repeatable AEP trace above background, and an FFT peak at twice the stimulus frequency.

At the end of the experiment and just prior to sacrifice by cervical transection, fish were measured for standard length (SL) and body mass (BM), and blood samples were collected from the caudal vein with 50 µl capillary tubes. Blood samples were centrifuged for 10 min at 8000 rpm and plasma was removed and stored at −80°C until analysis. Gonads were then removed to determine GSI.

### Steroid hormone assays

To test whether hearing thresholds were correlated with circulating sex steroid concentrations, we measured plasma levels of testosterone (T), 11-ketotestosterone (11-KT), and 17β-estradiol (E_2_) in all AEP animals at the end of the recording experiment with Enzyme ImmunoAssay kits (Cayman Chemical, Inc.) as previously described [39]. Hormone assays were validated previously for this species [39], extraction efficiencies were 89–92%, and intra-assay coefficients of variation were: T (10.1%); 11KT (6.8%); E_2_ (7.9%).

### Female preference experiments

To test whether sexually receptive gravid females used acoustic cues from courting males in their mate preference decisions, we simultaneously presented individual females with two visually similar males, one of which was previously associated with a sound playback while the other was not. An experimental aquarium (48×52×86 cm) was divided into three equal compartments with clear acrylic barriers, gravel covered the floors of the tank and one terra cotta pot was placed in each outer compartment to serve as a shelter and spawning territory for the males. Two different pairs of size and color-matched dominant males (SL = 76.5±1.7 mm; BM = 12.0±1.1 g) were selected from community tanks where they displayed typical dominance behaviors (e.g., chasing, courting, lateral displays) and coloration (eye-bar, anal fin egg spots, bright yellow body, red humeral patch) for 3 weeks prior to use in these experiments. Five trials of each sound type were performed with each male pair. One dominant male was placed into each outer compartment of the experimental tank along with a non-gravid female to facilitate his acclimation and territory establishment. Dominant males were given 48 hrs to acclimate to their new environment before their first behavioral trial.

To test whether females would prefer a male that was associated with natural conspecific courtship sounds over a control noise sound, we used playbacks of two different stimuli: 1) male courtship sounds, and 2) brown noise (control). The sound file of male courtship sounds was created from recordings from 3 different males of similar size that were strung together to create a 20 min sound file. Brown noise (spectral frequency of 1/f^2^, where f = frequency; decrease in intensity by 6 dB per octave) was chosen as a control because it contains higher energy at lower frequencies and lower energy at higher frequencies than white and pink noise, and thus is more similar in spectral content to natural male courtship sounds. Sound files were played back via a computer (Cool Edit Pro v2.1), amplified (TOA CA-160), and sent to the underwater speaker (UW-30) in the tank. Prior to experiments, we placed a hydrophone at various locations within the experimental tank and recorded the playbacks to verify that 1) sounds could be detected in the central compartment and were amplitude-matched between courtship and control noise sound files, 2) sounds could not be detected in the compartment of the male on the opposite side of the tank, and 3) playback sound frequencies were much lower than the minimum resonance frequency of the tank (calculated as 3.6 kHz according to equations in [54]) and did not show any obvious distortions from the original file.

Mate preference trials were all performed at the same time of day (0900-1100) to minimize any diurnal differences in female motivation or male behavioral displays. A gravid sexually receptive female (SL = 51.3±1.1 mm; BM = 3.65±0.24 g; GSI = 9.32±0.05; *N* = 10 fish per sound playback type) was obtained from a community tank on the morning of each experiment, and was visually selected based on a distended abdomen prior to morning feeding (a proxy for high GSI). Prior to the start of the trial, opaque barriers were placed alongside the transparent barriers to block the gravid female's view of both males and the speaker during the playback period. Non-gravid females were also removed from the outer compartments to ensure that the males interacted only with the focal gravid female during the experimental trial. The underwater speaker was placed in one of the outer compartments facing the central compartment, and then the focal gravid female was placed in the central compartment. The central compartment was divided into 3 zones for the purpose of later behavioral analysis; a ‘neutral zone’ in the center, flanked by ‘preference zones’ on either side that were marked within 7.5 cm of the side acrylic barriers. Fish were allowed to acclimate for 5–10 min before a sound stimulus was played. Sound stimuli, either brown noise or courtship sounds, were then played to the gravid female for 20 min. After the 20 min playback, the speaker and opaque barriers were removed so that the gravid female in the central compartment could see and interact with both of the dominant males in the outer compartments. This experimental setup meant that the females were presented with the sound stimuli without any visual cues from the males. This was necessary to avoid any preferences or avoidance to the large underwater speaker itself, and to eliminate any mismatches between sound playback and visual cues from male behaviors. Both the stimulus presentation period (20 min) and the post-stimulus period (35 min) were video recorded (Canon FS21). Each trial was randomized in terms of which male was affiliated with the playback and which sound type was played (courtship or noise control). At the end of the 35 min preference trials, the gravid female was anesthetized, sacrificed, and measured for SL, BM, and GM as described above for the AEP experiments. Thus, each female was used only once, and those with GSI values<6.0 were excluded from all analyses. There was no difference in SL, BM or GSI between females used in courtship sound versus control noise playback trials (t-tests, *p*>0.05).

To determine whether gravid females preferred to affiliate with the sound playback side versus the no sound side, we quantified behaviors of the subject female, as well as both of the dominant males only during the 35 min period following stimulus presentation. Behavioral quantifications were performed blind without knowledge of the side associated with sound, nor the sound playback type. For subject gravid females, we measured affiliation as the total time she spent with >50% of her body within each ‘preference zone’. All females included in the analyses spent time in both preference zones. To account for any effects of male behaviors on female preference that might not be related to sound playback, we also quantified the number of courtship quivers performed by each of the two males and then used these data to calculate a female ‘preference index’ for each trial. First, a relative preference ratio (RPR) was calculated for the sound side and the no sound side as: RPR = (percentage of time female spent in preference zone)/(number of quivers performed by male associated with preference zone). Preference index (PI) was then calculated as: PI = (RPR for the male associated with sound – RPR for male not associated with sound)/(RPR for male associated with sound + RPR for male not associated with sound). This gave us a PI between 1 and −1, with a positive value indicating a preference for the male associated with sound playback and a negative value indicating a preference for the male associated with no sound. This relative preference index methodology was similar to that used previously to test female preferences for courtship sounds in several Lake Victoria cichlids [25].

### Statistics

Linear regression was used to test for relationships between sound characteristics and male body size. To test for differences in hearing thresholds and circulating sex steroid levels, we used general linear mixed model repeated measures ANOVAs with thresholds for each of the 11 test frequencies or steroid levels for each of the 3 hormones as repeats (within-subject factors) and reproductive state (females) or social status (males) of the animal as the between-subject factor. Student's t-tests were used to compare GSI values between reproductive states within each sex. To test for correlations between hearing threshold and circulating sex steroid levels, we used Pearson Product Moment tests. Female preference data were compared with Student's t-tests. Data that did not meet the assumptions for parametric statistics were transformed (log, square root) prior to testing. Statistical analyses were performed with SigmaPlot 11.0 (Systat, Inc., San Jose, CA.) and SPSS 19.0 (IBM Corp., New York).

## Results

### Male sound production during courtship: behavior and sound characteristics

**Figure 1:**
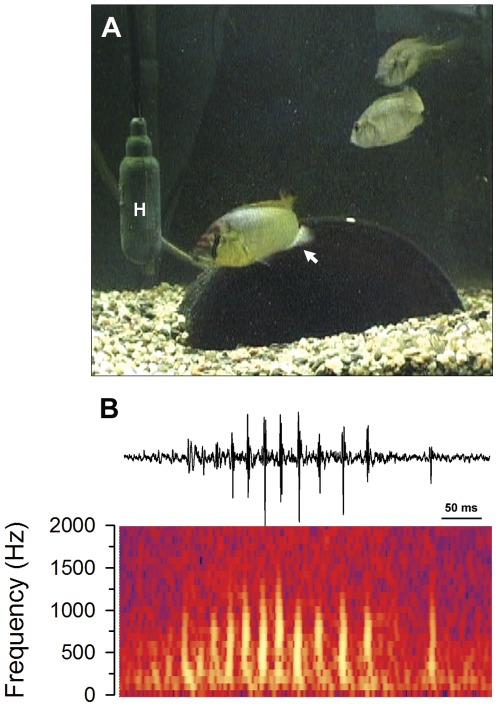
Dominant male *Astatotilapia burtoni* produce pulsed sounds during courtship quiver behaviors towards females. A) Photograph of a yellow dominant male in front of his pot territory performing a quiver display and courtship sound while presenting his anal fin egg-spots (arrow) towards two nearby, and attentive, gravid females. H, hydrophone. B) Representative waveform (top) and spectrogram (bottom) of a pulsed broadband courtship sound produced by a dominant male.

**Figure 2:**
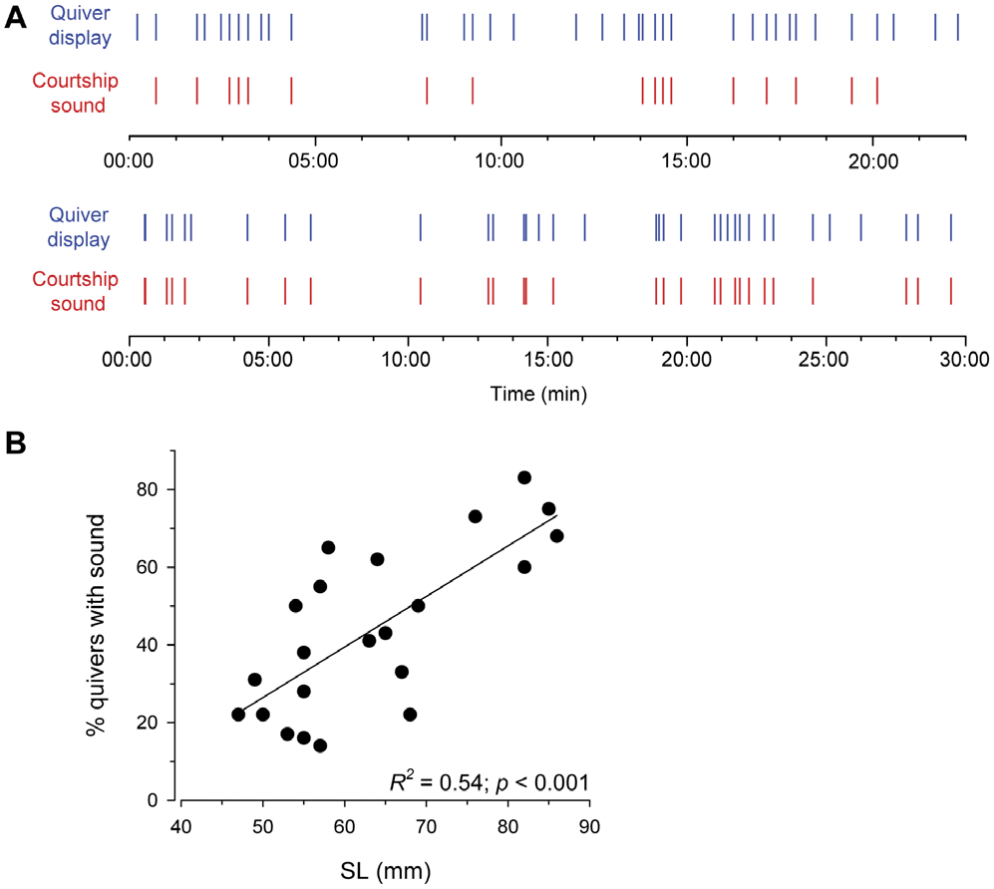
Dominant male *A. burtoni* produce intentional sounds during courtship quivers. A) Examples of the temporal sequence of courtship sounds and quiver behaviors produced by two individual males of different sizes. Top graph shows a small male (SL = 55 mm) that produced courtship sounds during ∼40% of behavioral quiver displays, while the bottom graph shows a larger male (SL = 82 mm) that produced sounds during ∼80% of quivers. Each vertical mark represents a single courtship sound or quiver behavior during the 30 min trial. B) Relationship between the percentage of quiver behaviors associated with courtship sounds and male standard length (SL) shows that larger males produce a greater proportion of behaviors with sounds than do smaller males.

Dominant males produced sounds during courtship quiver displays, and occasionally during tail waggles associated with leads towards the spawning territory. These quivers were defined by a flexion of the body associated with rapid movement and shaking of primarily the caudal portion of the body and tail with simultaneous presentation of the egg-spot-containing anal fin towards nearby females (Fig. 1; Video S1, S2, S3). Twenty-two different males were watched for a total of 569 min, and of that time, <1% was spent actually performing the rapid courtship quivers that are associated with the sound trains (i.e., most quivers are ≤1 sec in duration). Sounds were also produced during quivers at all stages of courtship, including immediately prior to spawning. Importantly, however, while all sounds were associated with behavioral displays, not all quivers or tail waggles were associated with sound production (Fig. 2). There was also a positive linear relationship between the percentage of these quiver behaviors associated with sounds and male body size (*R^2^* = 0.54, *p*<0.001) (Fig. 2).

**Figure 3:**
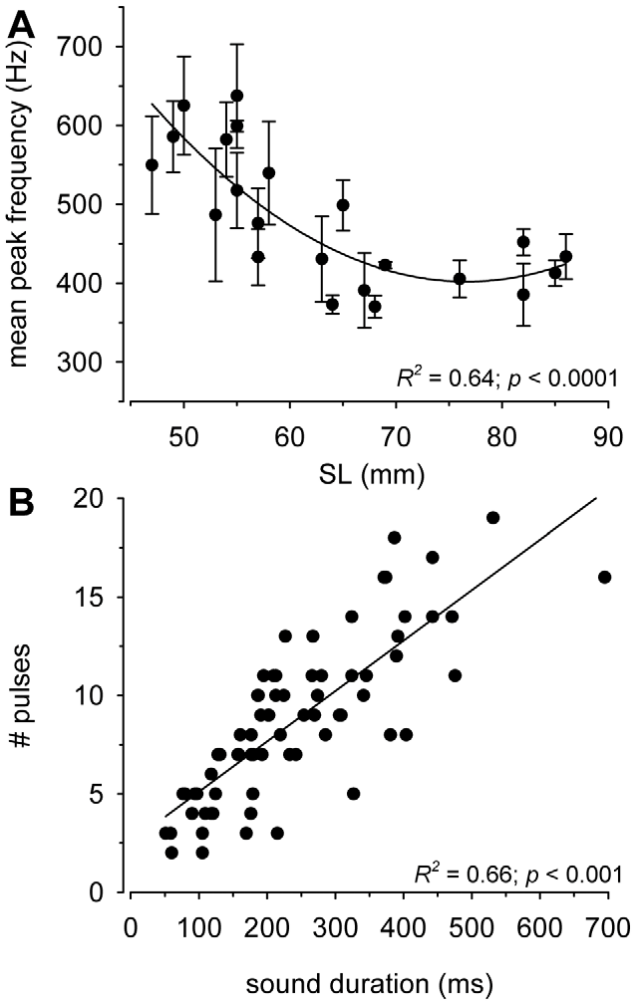
Characteristics of courtship sounds produced by male *A. burtoni* during quiver behaviors. A) Relationship between mean peak frequency (Hz) of sounds and male body size (standard length) shows that larger males produce lower frequency sounds. Each point represents the mean±SE of several sounds produced by an individual fish. B) There is a positive linear relationship between the number of pulses per sound and total sound duration (ms).

Courtship sounds (∼50–700 ms duration) consisted of a train of short (∼10–20 ms) pulses (∼8 pulses per sound) primarily produced as the male quivered his body and presented his anal fin egg-spots towards a nearby female (Fig. 1). Sound characteristics are summarized in Table 1. Power spectra of these sounds were relatively broadband (<50–1500 Hz) (Fig. 1), and there was a negative relationship between mean peak frequency and male body size (*R^2^* = 0.64, *p*<0.0001) (Fig. 3). There was also a positive relationship between total sound duration and the number of pulses within each sound (*R^2^* = 0.66, *p*<0.001) (Fig. 3).

**Table 1:** Summary of characteristics of courtship sounds produced by dominant male *Astatotilapia burtoni*.

	*N*, *n*	Mean±SD	Range
# pulses per sound	22, 74	8.5±4.1	2–19
Sound duration (ms)	22, 74	239.5±136.8	51.4–694.9
Pulse duration (ms)	22, 378	10.4±3.2	4.5–26.4
Interpulse interval (ms)	22, 366	18.3±13.0	5.3–97.5
Peak frequency (Hz)	22, 378	499.1±160.4	129–904

*N*, number of animals; *n*, number of sounds or pulses analyzed.

### Hearing ability: auditory evoked potentials

**Figure 4:**
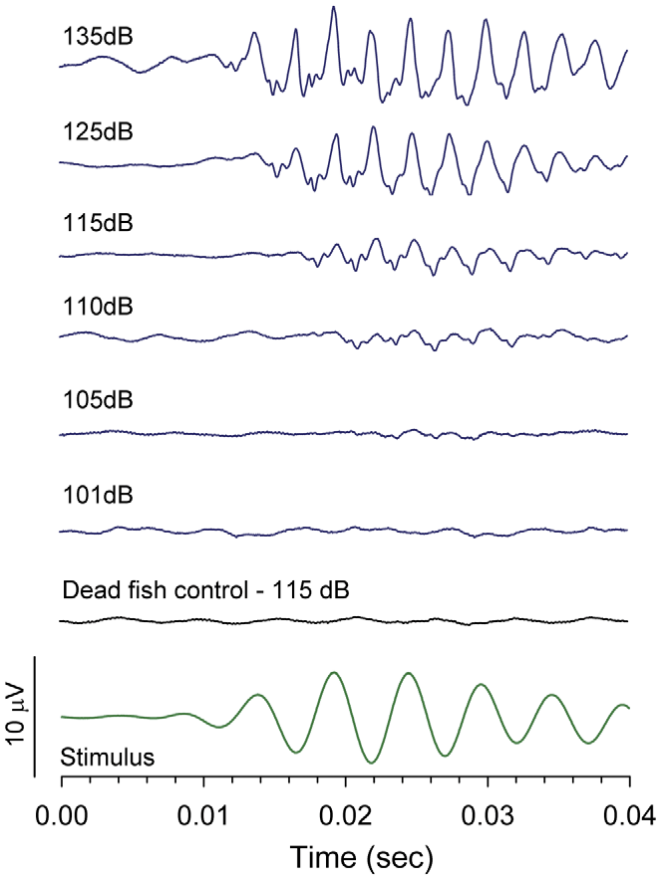
Example of auditory evoked potential (AEP) traces recorded from *A. burtoni*. Averaged AEP traces from a representative subordinate male in response to a 200 Hz stimulus at several different intensities. An averaged trace from a control dead fish at 120 dB in response to a 200 Hz stimulus shows no response. Bottom trace shows the actual stimulus waveform recorded by the hydrophone at the position of the fish head. Threshold at this frequency was 105 dB_rms_ re: 1 µPa based on the repeatable waveform and the presence of an FFT peak at twice the stimulus frequency.

Auditory evoked potentials were obtained from all males and females, and averaged response traces within a frequency were similar among all individuals tested. Representative averaged AEP traces from an individual male are shown in Fig. 4.

**Figure 5:**
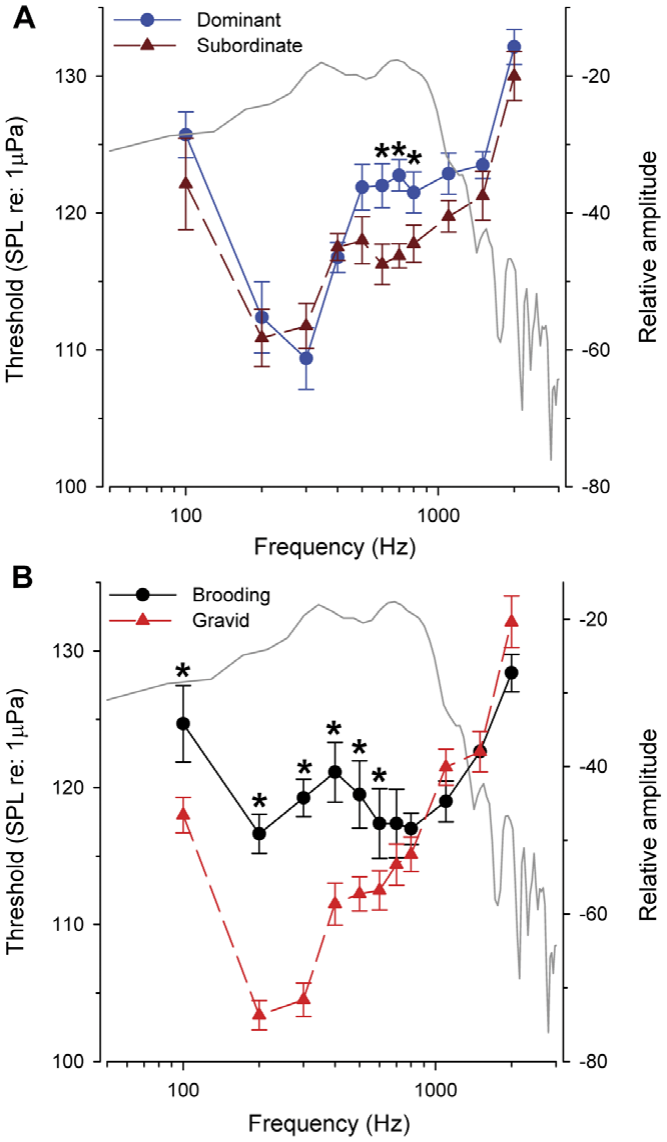
Hearing thresholds in the cichlid fish *A. burtoni*. A) Hearing thresholds for subordinate and dominant males show similar responses, but subordinate males had lower thresholds at 600–800 Hz. B) Hearing thresholds for females show that receptive gravid individuals have lower thresholds at low frequencies from 100–600 Hz compared to mouthbrooding females. Threshold data are plotted as mean±SE (left axis). Asterisks indicate statistical differences between reproductive states within a sex at each test frequency (*p*<0.05). Gray overlay lines represent the power spectra (128 point FFT, Hanning window) of a representative courtship sound and are plotted as relative amplitude in dB (right axis) for comparison of sound spectral energy to hearing thresholds. *N* = 8 fish for each reproductive state (for females) and social status (for males).

All fish showed repeatable AEP responses across all test frequencies from 100–2000 Hz. Mean auditory thresholds for all fish show that *A. burtoni* is most sensitive to low frequencies, with a best frequency at 200–300 Hz (Fig. 5). For both sexes, there was a 15–25 dB difference in threshold level between the frequency of best sensitivity (200–300 Hz) and worst sensitivity (2000 Hz). Subordinate males had lower thresholds at the higher frequencies of 600 to 800 Hz compared to dominant males (between-subject factor, F_(1,14)_ = 7.22, *p* = 0.018; 600 Hz, *p* = 0.019; 700 Hz, *p* = 0.001; 800 Hz, *p* = 0.044), but there was no difference in hearing threshold at any other frequency (*p*>0.05) (Fig. 5). In females, gravid individuals had lower thresholds (∼5–15 dB) at low frequencies from 100 to 600 Hz compared to mouthbrooders (between-subject factor, F_(1,14)_ = 13.99, *p* = 0.002; 100 Hz, *p* = 0.005; 200 Hz, *p*<0.001; 300 Hz, *p*<0.001; 400 Hz, *p* = 0.003; 500 Hz, *p* = 0.020; 600 Hz, *p* = 0.049), while thresholds at the higher frequencies (≥700 Hz) did not differ (*p*>0.05) (Fig. 5).

As expected, dominant males had GSI values two-fold greater than subordinate males (sub: 0.43±0.04; dom: 0.96±0.07; t-test, t = −6.56, *df* = 14, *p*<0.001), and gravid females had GSI values ten-fold higher than brooding females (br: 0.79±0.15; gr: 7.7±0.65; t-test, t = −10.32, *df* = 14, *p*<0.001). Dominant males also had higher circulating levels of T, 11-KT and E_2_ compared to subordinate males (between-subject factor, F_(1,14)_ = 34.92, *p*<0.001; 11-KT, *p* = 0.018; T, *p* = 0.008; E_2_, *p* = 0.001), while gravid females had higher levels of circulating T and E_2_, but not 11-KT, compared to mouthbrooding females (between-subject factor, F_(1,14)_ = 9.27, *p* = 0.009; 11-KT, *p* = 0.405; T, *p* = 0.010; E_2_, *p* = 0.011).

Hearing thresholds were correlated with circulating sex steroid levels in both males and females, but in opposite directions (Table 2). In males, there was a positive correlation between hearing threshold at 200 Hz and plasma levels of 11-KT and T, but not E_2_. Conversely, in females, there was a negative correlation between hearing threshold at 200 Hz and plasma levels of both T and E_2_, but not 11-KT. Higher GSI was also correlated with lower hearing thresholds (greater sensitivity) in females, but not in males (Table 2).

**Table 2:** Correlations between auditory evoked potential hearing threshold, circulating sex steroid levels, and gonadosomatic index (GSI) in the African cichlid fish *Astatotilapia burtoni*.

	T		11-KT		E_2_		GSI	
	*r*	*p*	*r*	*p*	*r*	*p*	*r*	*p*
**Threshold at 200 Hz:**								
**Males**	0.57	**0.03**	0.59	**0.02**	0.04	0.89	0.26	0.33
**Females**	−0.67	**0.007**	0.01	0.96	−0.60	**0.01**	−0.85	**<0.001**

11-KT, 11-ketotestosterone; E_2_, 17β-estradiol; T, testosterone; *r*, correlation coefficient; *p*<0.05 are in bold.

### Female preference experiments

**Figure 6:**
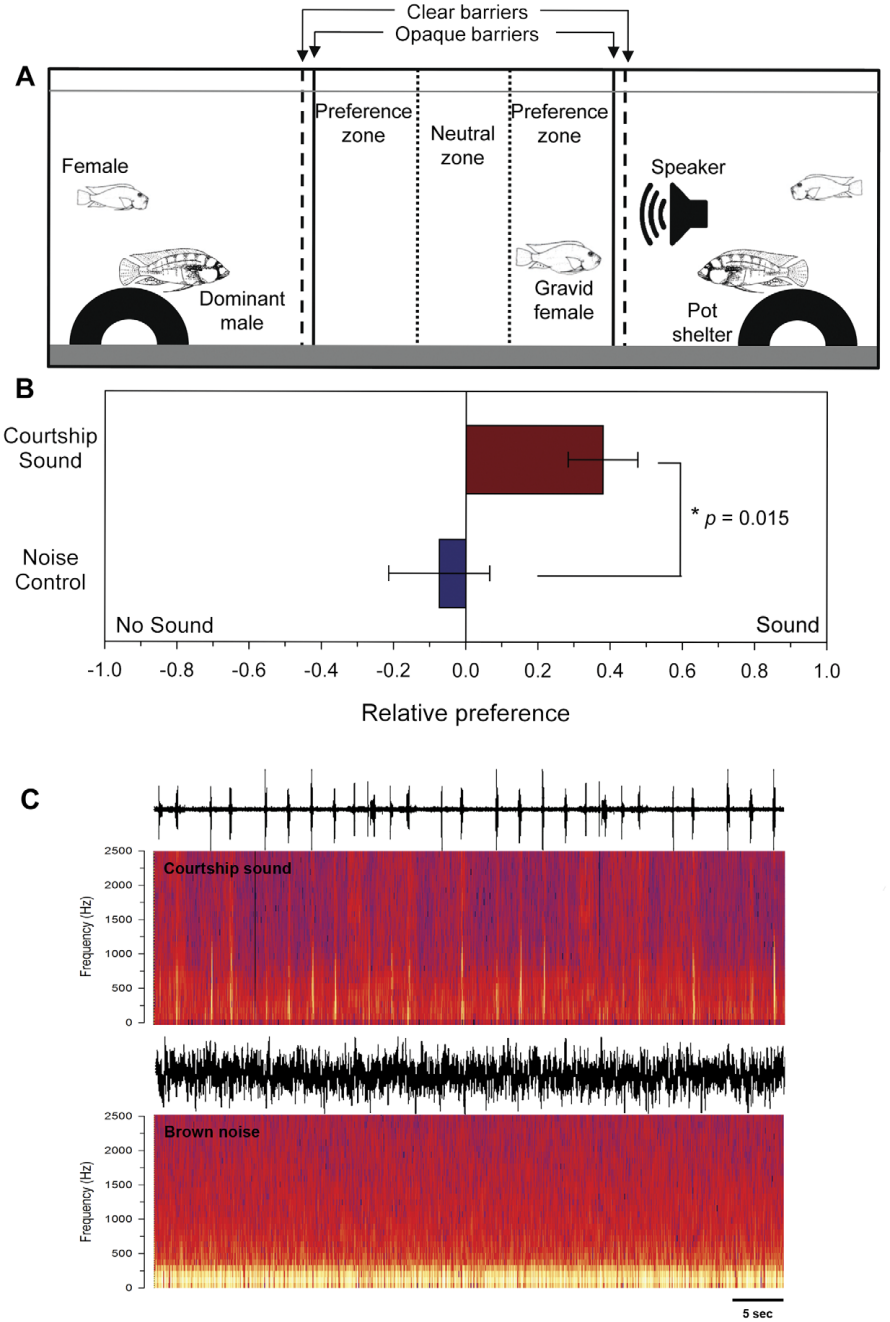
Gravid sexually receptive females prefer males associated with playbacks of courtship sounds over noise controls. A) Experimental tank setup for female preference trials. A gravid female was placed in the center compartment with size-matched territorial dominant males on either side. An underwater speaker in one of the male compartments played either male courtship or noise control sounds to the female while she was visually isolated. Following playback, the opaque barriers were removed and the time spent by the female in neutral and preference zones, as well as male reproductive behaviors were recorded and analyzed. B) Gravid females showed a greater relative preference (preference index) for males associated with the playback of courtship sounds compared to playbacks of control noise sounds. Data are plotted as mean±SE. *N* = 10 gravid females per sound condition. C) Examples of the playback stimuli used for courtship sounds and brown noise control trials. Waveforms (top) and spectrograms (bottom) for a 1 min section of each sound type are shown.

Gravid females spent more time in the preference zone of the male associated with playback of courtship sounds compared to the no sound side (t = 2.40, *df* = 18, *p* = 0.027). In contrast, there was no difference in the time females spent on the sound side versus the no sound side when control noise was played through the speaker (t = 0.33, *df* = 18, *p* = 0.743). When the activity of the males was taken into account (see methods), gravid females preferred males that were associated with playbacks of courtship sounds over noise controls (t = −2.67, *df* = 18, *p* = 0.015) (Fig. 6).

## Discussion

Here we used a multidisciplinary approach to test the hypothesis that the Tanganyikan cichlid *A. burtoni* uses acoustic communication as part of its courtship behavior. Our results demonstrate that dominant males produce courtship sounds in proximity to females as part of their reproductive repertoire, and that sounds are spectrally similar to their hearing ability. Further, we show that receptive gravid females are 2- to 5-times more sensitive to low frequency sounds compared to mouthbrooding parental females, which may facilitate detection of the spectral components of male courtship sounds when they are ready to spawn. Hearing thresholds were correlated with circulating sex steroid levels in both males and females, although in opposite directions, suggesting a potential role for sex steroids in their reproductive state-dependent auditory plasticity. Behavioral experiments also showed that gravid females preferred to affiliate with males that were associated with playback of courtship sounds compared to noise controls, suggesting that acoustic information is used during female mate choice. Taken together, our results indicate that acoustic communication is important during reproduction in this species as part of a multimodal signaling repertoire. These data also suggest that perception of auditory information changes throughout the reproductive cycle, potentially mediated by gonadal state and circulating sex steroids. Our results also highlight the significance of examining non-visual signaling during context-specific behaviors in this speciose and evolutionarily valuable group of fishes.

### Sound production and behavior

Dominant male *A. burtoni* produced pulsed broadband sounds during body quivers associated with courtship behaviors. Our simultaneous sound and video recordings demonstrate that these courtship sounds are produced intentionally because not all quiver behaviors were associated with sound production, suggesting that the sound is not merely a by-product of body movements, but that males have some control over when and where it is produced. This is further supported by the fact that larger males were more likely to produce a sound along with their quivers, suggesting that male experience or age may play a role in acoustic communication. Sounds were also made primarily in close proximity to females, and were of relatively low intensity, indicating that they could only function, and hence are likely intended, for close-range communication. These quiver body movements also likely produce strong hydrodynamic components that could be detected by the female's lateral line system. The temporal (e.g., sound duration, pulse duration, number of pulses) and spectral (peak frequency) characteristics of *A. burtoni* courtship sounds were also similar to those previously described in this [23], [40] and other cichlid species (reviewed in [21], [55], [56]). The mechanism of sound production in *A. burtoni* is not known, however, it may involve the pharyngeal jaws and swimbladder as proposed for other cichlids [57] or be similar to that described for the related cichlid *O. niloticus* where a backward movement of the pelvic and pectoral girdles and forward movement of the anal fin is associated with contraction of bundles (vesica longitudinalis) in the axial musculature that compresses the rib cage and swimbladder to help produce the sound [58].

While interest in African cichlid sound production has increased in recent years, the majority of these studies examine species from Lake Malawi, Lake Victoria, and river systems [21], [22], [56], with little focus on Tanganyikan cichlids. Lake Tanganyika is the oldest, deepest, and most morphologically and behaviorally diverse of the rift lakes, and may have originated the cichlid radiation that gave rise to species in the other rift lakes [10]. Thus, understanding the role of acoustic and multimodal communication in species from Lake Tanganyika is essential to fully appreciate the driving forces, mechanisms, and pathways of diversification in cichlids.

### Hearing abilities

*Astatotilapia burtoni* was most sensitive to low frequencies from ∼200–600 Hz, with a best frequency at 200–300 Hz, which overlaps the spectral content of the courtship sounds produced by dominant males. While many studies have described sound production and associated behaviors in different cichlids [9], [17], [19], [20], [22], [23], [24], [25], [56], [58], [59], [60], [61], [62], hearing abilities have been examined in only a few species (e.g., *Tramitichromis intermedius* [48], *Astronotus ocellatus* [63], [64], *Tilapia macrocephala* [65], [66], *Neolamprologus brichardi* [67], and *Oreochromis niloticus* [68]). Further, the majority of these studies only tested the cichlid species as an example of a fish that does not possess specialized auditory structures (e.g., Weberian ossicles), for comparison to those that do (e.g., goldfish), rather than specifically to examine the biological significance of their hearing abilities. In fact, aside from *N. brichardi* being used as a goldfish comparison [67], ours is the first study, to our knowledge, to describe hearing abilities in any cichlid from Lake Tanganyika. As a result, little is known about how sound production is matched to hearing abilities in cichlid fishes, but along with the present study, there is evidence for this matching of low frequency sound production and hearing ability in *T. intermedius* and *Oreochromis* species [48], [62], [68], which highlights the potential importance of acoustic signaling in cichlid communication. However, it is also relevant to mention that exact matches in spectral content between hearing ability and sound production are not required for effective acoustic communication, as many sound-producing fishes show only weak correlations between best frequencies of hearing and dominant frequencies of sound production [69], [70]. This may be partially due to the fact that many fishes produce broad band sounds that contain multiple frequency components, so that sensitivity to a pure tone stimulus can be worse than to a multi-frequency complex sound with equal peak intensity but more total energy within a critical hearing band [71], [72]. Thus, the selective pressures acting on both hearing ability and sound production within a species are complex and deserve future study before generalizations on these aspects of acoustic communication among different taxonomic groups of fishes can be made.

To our knowledge, this is also the first study to show reproductive state differences in hearing ability in any cichlid fish. Gravid female *A. burtoni* had lower hearing thresholds compared to mouthbrooding females, and this improved sensitivity was correlated with higher GSI and higher circulating levels of T and E_2_. A previous study in *A. burtoni* also showed that mRNA levels of androgen and estrogen receptors in the saccule of the inner ear were lower in gravid females, and negatively correlated with circulating sex steroids [39]. This suggests that the peripheral auditory system changes throughout the reproductive cycle of females, and may be modulated by gonadal steroids. Female *A. burtoni* breed year-round and following release of their fully developed fry, undergo ovarian recrudescence and increases in circulating sex steroid levels over the next several weeks in preparation for the subsequent spawning cycle, a time course that suggests any changes in hearing ability could be mediated by both genomic and non-genomic mechanisms. A similar situation occurs in the seasonally breeding midshipman fish *Porichthys notatus*, where females in the breeding season have lower hearing thresholds and are more sensitive to the higher frequency components of the nesting males' advertisement calls compared to non-reproductive females [26], [73], [74], an auditory phenotype that can be replicated with T and E_2_ implants [26]. Moreover, changes in hearing ability associated with the female reproductive cycle and circulating hormone levels occur in many vertebrate taxa, including humans [27]. Some potential mechanisms that may be involved in the reproductive state auditory plasticity in *A. burtoni* include changes in central auditory processing in the brain, or variations at the periphery of the inner ear such as changes in hair cell numbers, ionic composition, or expression of ion channels [27], [30], [73], [74], [75], [76].

Subordinate male *A. burtoni* also showed lower hearing thresholds at frequencies from 600 to 800 Hz compared to dominant males. We speculate that improved hearing at these frequencies near the upper spectral range of male courtship sounds could allow subordinate males, which often school with females, to better locate territories of smaller dominant males (e.g., that produce sounds with higher frequency components) where they would have a greater chance of winning a challenge with the resident and acquiring his territory. Improved sensitivity may also allow these subordinate males, which typically have minimal spawning opportunities without a territory, to detect when a territorial dominant male is close to spawning so that he can capitalize on the chance to sneak spawn [77]. This ‘interception’ function also occurs in other vocal fishes such as the midshipman *P. notatus*, where both females and sneaker males show positive phonotaxis to playbacks of advertisement calls from nesting males, suggesting that both sexes use auditory signals to localize spawning areas and reproductive opportunities [78]. Interestingly, we previously showed that subordinate males had higher mRNA levels of some estrogen and glucocorticoid receptor subtypes in the inner ear compared to dominant males [39], which may play a role in the improved hearing at these higher frequencies. Alternatively, the threshold differences at 600–800 Hz may function to detect other acoustic signals such as feeding sounds, aggressive sounds or predators, or simply be an artifact of the experimental setup or low sample size that requires further investigation.

### Role of male courtship sounds in female mate preference

Dominant male *A. burtoni* produced courtship sounds during body quivering displays in proximity to conspecifics (primarily females, but occasionally other males). The proximity to other individuals and the rapid attenuation of the sounds produced by signaling males suggests that sound production in *A. burtoni* is intended for close-range communication, and likely serves to advertise the presence, reproductive readiness/motivation, and quality of the male sender to the females. This has also been suggested for other cichlid species that produce similar courtship sounds during close-range quiver behaviors [9], [17], [24], [60], [79]. Importantly, these quivers associated with sound production provide a stimulus that can be detected by both the inner ear and mechanosensory lateral line system, but how this information might be differentially used by the female remains unknown. Since many of the acoustic characteristics associated with sound production are energetically expensive, they likely function as honest signals used during mate choice, as demonstrated in other vertebrate and invertebrate taxa [80], [81], [82]. However, it is also likely that other non-intended receivers, both males and females, in the vicinity of a courting male can eavesdrop on the sounds and use it to gain social, spawning, or feeding opportunities. Eavesdropping on acoustic signals has been described in many vocal taxa as a tactic to improve survival and reproductive fitness [83], [84], [85], and therefore may play a role in the natural selection of territorial cichlids as well.

Similar to the one other study on the role of acoustic signals during female mate preference in cichlids [25], and due to technical limitations, the gravid females in our experiment heard the courtship (or noise) sounds before they could see the males, thus the visual and acoustic signals were temporally uncoupled from each other. The sounds alone, therefore, influenced the female's preference before she acquired any visual cues (e.g., coloration, size, behaviors) from the male, suggesting that overhearing the sound production itself provides the female with some valuable information, such as advertising that a reproductively motivated male is in the area and is actively trying to entice females into his territory to spawn. Importantly, however, the inclusion of control noise playbacks in our study also demonstrates that female preference in *A. burtoni* is not simply a response to any sound, but is specific to the natural courtship sounds produced by males. This eavesdropping function is further supported by the improved hearing ability in females that are gravid and ready to spawn, which would allow them to detect courting males at greater distances, potentially resulting in increased reproductive fitness. Thus, this is also the first study to demonstrate that acoustic information is used for female mating preferences in a Lake Tanganyikan cichlid, which has important evolutionary implications (see below).

### Multimodal communication during courtship in cichlids and evolutionary implications

A previous study in *A. burtoni* showed that when females are gravid, they prefer to affiliate with dominant males over subordinate males, a preference that doesn't exist when they are in the non-gravid stage of their reproductive cycle [86]. While there are many visual cues that would allow females to distinguish male social status (e.g., coloration patterns, relative size, behaviors, territory quality), our results here now suggest that they likely also use auditory cues to gain information on potential mates. For example, in nature, females may use auditory signals to localize male territories, detect more active males based on the relative number of courtship sounds produced, and determine male size or other quality indicators based on the spectral and temporal characteristics of their sounds. The close-range quiver behaviors would also generate hydrodynamic cues that could be detected by the female's lateral line system, but how mechanosensory signaling might function in this species is not yet known. We do know, however, that chemical communication is important during reproduction in *A. burtoni* [38], [41], [87], and that perception of olfactory signals may also depend on female reproductive state [88]. Thus, the reproductive repertoire of this African cichlid involves multisensory signaling (e.g., visual, acoustic, chemosensory), which suggests that multimodal communication likely plays a more important role in mate choice decisions and sexual selection, potentially in many cichlids, than previously recognized (see [9]). Further, our study highlights the importance of including not only multimodal communication features in models of sexual selection, but also the plasticity of an animals' internal state (e.g., hormones that fluctuate with social status or reproductive condition) that can influence both signal output, as well as signal reception, across different spatial and temporal scales.

Previous studies have suggested that single traits are often insufficient to explain phenotypic diversity in cichlids and that species richness is a function of the number of traits involved in diversification (i.e., the ‘multifarious selection’ hypothesis) [18], [89], [90]. Thus, the use of multiple communication systems for reproduction provides more traits on which sexual selection can act, allowing for a greater number of taxa and resulting in the high diversity of cichlid fishes [10], [18]. Studies on a limited number of cichlids from Lake Malawi and Victoria, as well as riverine species such as *Oreochromis*, suggest that multimodal communication (visual, acoustic, chemosensory) is important during reproduction, but it had not yet been demonstrated for any cichlid in the oldest, but most phenotypically diverse rift lake, Lake Tanganyika. We now have evidence, however, that the Tanganyikan cichlid *A. burtoni*, a sister group to the Lake Victoria superflock, uses visual, chemosensory, and acoustic communication during reproduction [32], [38], [41], suggesting that sexual selection acting on multiple traits may contribute more to the high phenotypic diversity found in Lake Tanganyikan cichlids [91] than previously realized. Thus, addressing features of multimodal communication in a comparative context should be a valuable future area of research to understand the evolutionary mechanisms underlying the remarkable African cichlid diversification and speciation.

## Supporting Information

Video S1 **Sound production during courtship in the African cichlid fish** ***Astatotilapia burtoni*** **.** A yellow dominant male produces two distinct pulsed courtship sounds while quivering his body and presenting his anal fin towards nearby gravid females. A hydrophone is suspended in front of the pot shelter used by the dominant male as a territory and spawning area.
(MP4)

Video S2 **A yellow dominant male** ***A. burtoni*** **quivers his body and produces a pulsed courtship sound just prior to leading a gravid female into his pot shelter.** A hydrophone is suspended near the pot shelter.
(MP4)

Video S3 **Example of another dominant male** ***A. burtoni*** **producing a courtship sound during a quiver display in front of his shelter.** Gravid reproductively receptive females are present and a hydrophone is suspended near the pot shelter.
(MP4)
